# Polarized Exocytosis Induces Compensatory Endocytosis by Sec4p-Regulated Cortical Actin Polymerization

**DOI:** 10.1371/journal.pbio.1002534

**Published:** 2016-08-15

**Authors:** Jesper Johansen, Gabriel Alfaro, Christopher T. Beh

**Affiliations:** 1 Department of Molecular Biology and Biochemistry, Simon Fraser University, Burnaby, British Columbia, Canada; 2 Centre for Cell Biology, Development, and Disease, Simon Fraser University, Burnaby, British Columbia, Canada; UT Southwestern Medical Center, UNITED STATES

## Abstract

Polarized growth is maintained by both polarized exocytosis, which transports membrane components to specific locations on the cell cortex, and endocytosis, which retrieves these components before they can diffuse away. Despite functional links between these two transport pathways, they are generally considered to be separate events. Using live cell imaging, in vivo and in vitro protein binding assays, and in vitro pyrene-actin polymerization assays, we show that the yeast Rab GTPase Sec4p couples polarized exocytosis with cortical actin polymerization, which induces endocytosis. After polarized exocytosis to the plasma membrane, Sec4p binds Las17/Bee1p (yeast Wiskott—Aldrich Syndrome protein [WASp]) in a complex with Sla1p and Sla2p during actin patch assembly. Mutations that inactivate Sec4p, or its guanine nucleotide exchange factor (GEF) Sec2p, inhibit actin patch formation, whereas the activating *sec4-Q79L* mutation accelerates patch assembly. In vitro assays of Arp2/3-dependent actin polymerization established that GTPγS-Sec4p overrides Sla1p inhibition of Las17p-dependent actin nucleation. These results support a model in which Sec4p relocates along the plasma membrane from polarized sites of exocytic vesicle fusion to nascent sites of endocytosis. Activated Sec4p then promotes actin polymerization and triggers compensatory endocytosis, which controls surface expansion and kinetically refines cell polarization.

## Abstract 2

Maintaining a constant cell membrane area depends on a balance between endocytosis and exocytosis. This study shows that the yeast Rab GTPase Sec4p, a well-established regulator of polarized exocytosis, induces compensatory endocytosis by promoting cortical actin polymerization.

## Author Summary

Cells maintain a constant size by keeping a balance between the intracellular transport pathways that take membrane material to and from the cell surface. How that balance in membrane trafficking is attained, and by what mechanism(s), is poorly understood. Here, we analyzed these potential mechanisms and found that the yeast Rab GTPase Sec4p, a protein that regulates transport to the cell surface (polarized exocytosis), coordinates this function with the assembly of cortical actin patches, which initiate endocytosis and the compensatory recycling of membrane back into the cell. We tracked Sec4p on the plasma membrane and showed that it associates with actin patches at a specific time during their maturation. Mutations in *SEC4* or its regulatory genes disrupted actin patches and inhibited endocytosis. In addition, we showed that Sec4p directly binds and regulates the activity of the yeast WASp homolog Las17p, which, together with the Arp2/3 complex, regulates actin polymerization and actin patch assembly. Based on these results, we propose that Sec4p overrides an inhibitory step during actin patch assembly to spur on endocytosis. Sec4p thereby balances the delivery of material to the cell surface with the induction of compensatory endocytic recycling to maintain a constant cell size.

## Introduction

In specific secretory cell types, endocytosis compensates for the expansion of cell surface area caused by vesicular transport to the plasma membrane (PM) [1]. The coupling of endocytosis with exocytosis controls membrane expansion in Xenopus oocytes during cortical granule exocytosis [2] and after vesicle transport to the PM in both neurons and non-neuronal cells [3]. In polarized cell types, polarized exocytosis is also linked to a reciprocal endocytic event where membrane traffic to and from adherens junctions maintain epithelial apical-basal polarity by recycling junctional proteins [4]. These examples raise the more general question of whether cycles of exocytosis and endocytosis are directly coupled in all cell types.

In *Saccharomyces cerevisiae*, the first indication that vesicular transport to and from the PM is coupled was the finding that mutations in late secretory (*SEC*) genes, required for the final stages of exocytosis, also disrupt uptake of lucifer yellow by endocytosis [5]. More puzzling was the finding that *SEC* genes required for earlier events in exocytosis had weak or variable effects on endocytic uptake, suggesting that any block in exocytosis does not result in a commensurate inhibition of endocytosis. Although there are notable exceptions in which early *SEC* mutants do impact endocytosis [6–8], a recent phenomics analysis of trafficking mutants generally supports the notion that the late stage of exocytosis is particularly important for regulating endocytosis [8]. Specifically, the exocyst complex, which tethers post-Golgi vesicles to the PM during exocytosis, was described as a “network hub” for coordinating the two trafficking pathways at the cell surface [8]. These results suggest the possibility of a direct regulatory mechanism for integrating the late stages of post-Golgi exocytosis with endocytic internalization.

Like other late *SEC* genes, *SEC4* appears to affect both exocytosis and endocytosis [5]. *SEC4* encodes a Rab GTPase that moves with exocytic vesicles along actomyosin cables from the Golgi to sites of polarized growth on the PM within the budding daughter cell [9,10]. The dynamic movement of Green fluorescent protein (GFP)-Sec4p particles during exocytosis has been tracked in living cells and involves two distinct patterns of motion: the first corresponds to the actomyosin-directed polarized transport of vesicles into the bud; the second follows membrane fusion of exocytic vesicles with the PM, during which GFP-Sec4p particles dwell briefly at the cell cortex, making small random movements before disappearing [9,11]. At first glance, Sec4p and its regulation of exocytosis appears to be completely independent from mechanisms controlling endocytosis. Given that the GDP dissociation inhibitor Gdi1p mediates Sec4p removal from the PM and trafficking back to the Golgi, even the recycling and internalization of Sec4p does not require endocytosis [12–14]. Nonetheless, it has been shown that Sec4p localizes to recycling endomembranes involved in establishing cell polarization [15]. This result hints that Sec4p might have an involvement in some aspect of endocytosis apart from its established role in polarized exocytosis.

In budding yeast, the polarized sites where exocytic vesicles dock at the PM partially overlap with nascent sites of endocytosis, which correspond to cortical actin patches [16–18]. The maintenance of polarized growth at these sites is dependent on both polarized exocytosis and endocytosis [19]. Classic yeast ultrastructural studies showed that exocytic vesicles are in close proximity to cortical actin but do not directly associate [16,17]. However, as polarized cell growth proceeds during the yeast cell cycle, exocytic docking sites and actin patches relocate in lockstep: first from the incipient bud site in unbudded cells to the bud tip in small-budded cells, then around the entire bud cortex in medium-budded cells, and finally to the mother/bud neck in large-budded cells. Even though exocytic vesicles do not directly interact with nascent sites of endocytosis, a specific regulator of exocytosis (like Sec4p) could laterally diffuse through the PM to interact with endocytic components at actin patches.

The generation of cortical actin patches for endocytic membrane internalization involves a series of distinct events, each punctuated by the formation and dissolution of specific protein complexes [18]. The duration of the initiating event is variable and relatively long (~1–2 min), which might represent a stochastic activation. During this phase, early coat proteins such as clathrin, Ede1p, and Syp1p are recruited, and computational modeling suggests that the packaging of membrane cargo helps drive this event forward [20,21]. Over the next 20–30 s, Sla1p, Sla2p, and Las17p are recruited during the slow coat assembly phase, which lays the groundwork for subsequent dynamic phases of actin network assembly [18]. Sla1p and the yeast HIP1r homologue Sla2p represent conserved clathrin adaptors, whereas Las17p is the yeast Wiskott—Aldrich syndrome protein (N-WASp) homologue [18,22–24]. Las17p is a potent activator of Arp2/3-dependent actin polymerization, but it is kept inactive during this period in part by an inhibitory interaction with Sla1p [24]. In this way, Sla1p plays dual roles during endocytic internalization, in which it acts both an activator required for coat assembly and an inhibitor by regulating the timing of subsequent steps in actin patch maturation [23–25]. The regulatory mechanism that frees Las17p from Sla1p inhibition is unknown, but it represents a key transitional mechanism for unleashing the rapid sequence of final events. In effect, this pause helps coordinate the completion of coated pit maturation with the onset of actin nucleation. Finally, during the next actin patch assembly phase, the inward force for membrane internalization is provided. This step is characterized by rapid patch dynamics, as reflected in the motility of Abp1p [26], a mediator of Arp2/3-dependent F-actin branching [27]. Inward PM deformation is quickly followed by membrane scission, which completes membrane internalization and the biogenesis of endocytic vesicles.

Here, we investigate if a direct regulatory mechanism exists to integrate polarized exocytosis with endocytosis. We show that the polarized delivery of Sec4p regulates the stage-specific assembly of actin patches by promoting actin polymerization. In addition to being a well-established regulator of exocytosis, Sec4p met all the expectations for a regulator of endocytosis: (i) Sec4p transiently localized to endocytic sites at a specific period during their formation; (ii) *SEC4* mutations disrupted actin patch assembly; (iii) Sec4p physically interacted in vitro and in vivo with Las17p, which is required for cortical actin nucleation; (iv) in the presence of Las17p and Sla1p, Sec4p induced Arp2/3-dependent actin polymerization in vitro; (v) Sec4p polarization contributed to the polarized assembly of actin patches. These results indicated that Sec4p overrides a limiting step in actin patch formation and thereby directly couples transport cycles to and from the cell cortex.

## Results

### Sec4p Is Recruited to Nascent Cortical Actin Patches to Promote Their Assembly

Targeting and docking of exocytic vesicles to the PM is mediated by the multi-protein exocyst complex. Sec4p anchors a subset of exocyst complex subunits, including Sec5p, to the vesicle membrane [28]. Even though Sec4p and Sec5p travel together to the PM [10], we observed that at the cortex the actin patch marker Sla1p-Red fluorescent protein (RFP) co-localized four times more often with GFP-Sec4p particles than with Sec5p-3xGFP particles (*n* > 340; *p* < 0.0001). This result suggested a link between Sec4p and nascent sites of endocytosis as defined by actin patches, distinct from other subunits of the exocyst complex and exocytic vesicles. However, there are several criteria to be met if Sec4p is proposed to play a direct role coordinating actin patch assembly with its established role during the late stages of exocytosis. First, because of the hierarchal addition of actin patch subunits, if Sec4p is a direct regulator of patch assembly, it must exhibit both a specific spatial and temporal co-localization with nascent endocytic sites. Second, as readouts of actin nucleation and network formation at patches, actin patch dynamics and/or morphology would be disrupted as a result of a genetic defect in any regulator of endocytic internalization. If Sec4p coordinates endocytic internalization with exocytosis, then we also predict that Sec4p motility at the PM would be affected by defects in actin patch assembly and vice versa. If Sec4p represents the mechanism by which polarized exocytosis promotes actin patch polarization, then changes in Sec4p activity and distribution would be predicted to affect the asymmetric formation of actin patches within budding cells. Finally, a direct role in endocytic internalization necessitates that Sec4p physically interacts with specific actin patch subunits to regulate a particular mechanistic event during patch assembly.

**Fig 1:**
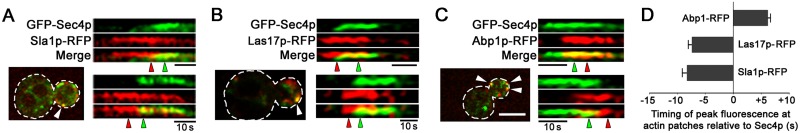
Spatial and temporal co-localization of Sec4p with actin patch subunits during actin patch assembly. **A–C.** Images of wild-type cells (WT; BY4741) showing the co-localization (arrowheads) of newly transported GFP-Sec4p particles after photobleaching with Sla1p-, Las17-, and Abp1-RFP (bar = 2 μm). Duplicate examples of kymographs are shown comparing the relative timing of GFP-Sec4p co-localization with each actin patch subunit. For each kymograph shown, green (GFP-Sec4p) and red (RFP fusions) arrowheads indicate maximum voxel fluorescence intensities. **D.** Bar graph reporting average differences in time for the maximum fluorescence of each RFP-marked actin patch subunit relative to GFP-Sec4p (*n* = 22 particles/strain; kymographs from ≥ 11 independent cells). In all graphs, data is shown as mean values with error bars representing standard error of the mean (S.E.M).

As both a test of our criteria and to establish if Sec4p directly regulates endocytosis, GFP-Sec4p localization at the PM was analyzed and the effect of inactivating *SEC4* on actin patch assembly was examined. Because of the fleeting nature of actin patch dynamics, the temporal and spatial overlap between GFP-Sec4p and actin patches was most evident when individual GFP-Sec4p particles were tracked on the cell cortex (Fig 1). The GFP-Sec4p fusion construct used for these analyses is an established reporter of Sec4p in yeast, as shown by immunofluorescence and immunoelectron microscopy [9]. Nevertheless, individual GFP-Sec4p particles are difficult to visualize in living cells because of the continuous transport of exocytic vesicles to the PM, where GFP-Sec4p appears at the bud cortex as a “churning mass” [9]. To track individual GFP-Sec4p particles, we focused our analysis on medium-to-large budded cells in which GFP-Sec4p particles were less densely distributed on the PM. We also used fluorescence photobleaching to track newly transported GFP-Sec4p particles on the cell cortex (S1 Movie). In these experiments, actin patch assembly and vesicle trafficking, which are inherently readouts of cell growth, continued as normal. Moreover, there were no adverse effects on cell viability even when the laser intensity and the duration of photobleaching were both increased 10-fold beyond that used in these experiments (see Materials and Methods). For all fluorescent markers tested (GFP-Sec4p and Abp1p-, Sla1p-, and Las17p-RFP), particle lifetimes were equivalent in photobleached cells as compared to those that were not (by one-way analysis of variance at a 95% confidence limit; *n* ≥ 36 cells). Thus, both the photobleaching conditions and the GFP-Sec4p reporter itself were suitable for examining dynamic associations between individual GFP-Sec4p particles and assembling actin patches.

To analyze GFP-Sec4p particles during endocytic site formation, GFP-Sec4p was visualized by confocal fluorescence microscopy relative to the defined timing of subunit recruitment during actin patch assembly [18,26]. After their transport into photobleached zones at the bud cortex, >64% of individual GFP-Sec4p particles co-localized with Abp1p-, Sla1p-, and Las17p-RFP foci (*n* > 90). Kymograph analysis of GFP-Sec4p localization during actin patch assembly also showed an invariant timing; Sec4p always associated after Sla1p and Las17p recruitment, and then Sec4p disappeared immediately following the arrival of Abp1p (Fig 1A–1D, S2–S4 Movies). In short, this analysis showed that Sec4p and proteins involved in endocytic internalization concurrently localize during a specific stage in actin patch assembly.

**Fig 2:**
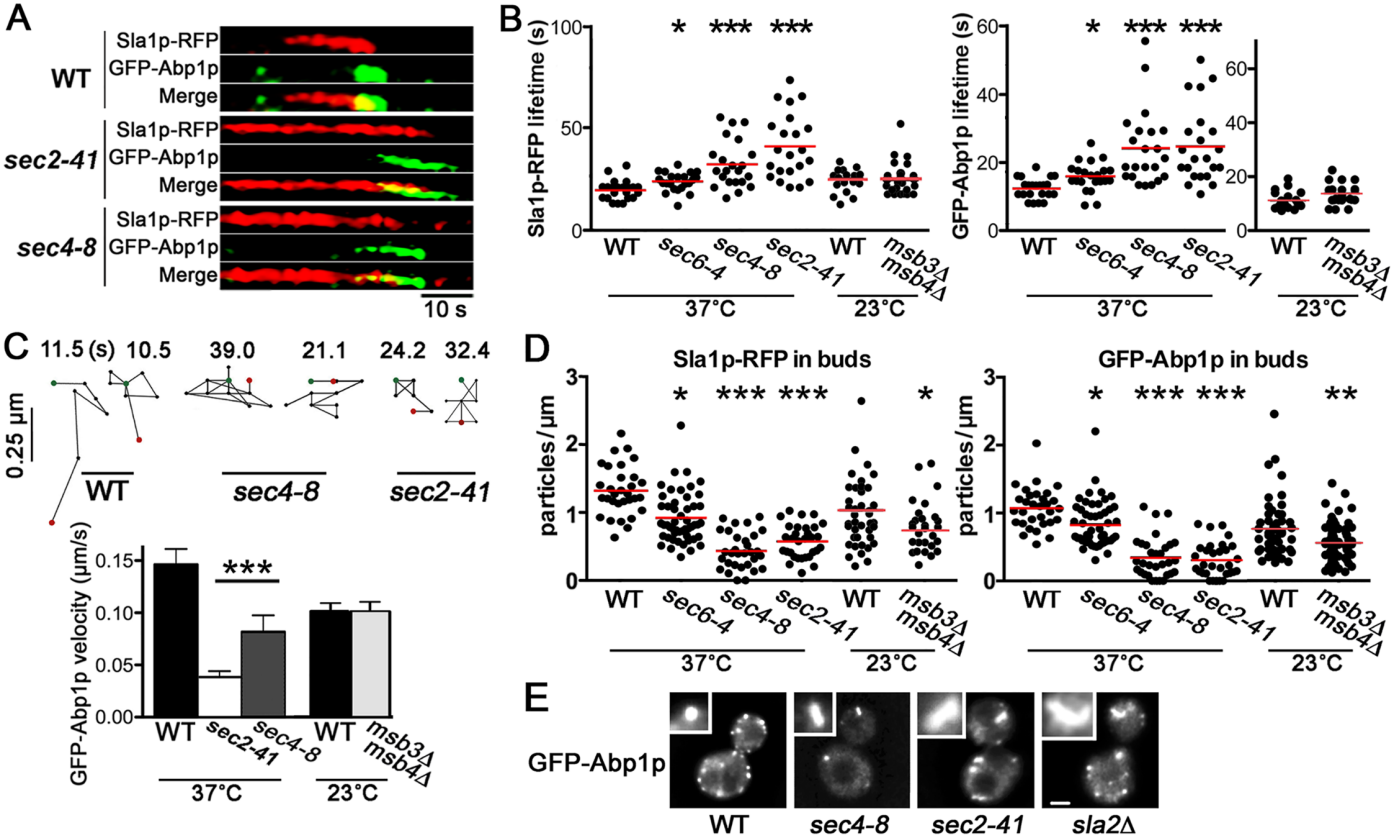
Mutations in *SEC4* and *SEC2* disrupt actin patch assembly and proper endocytic internalization. **A**. Kymographs show coincident localization of single Sla1p-RFP and GFP-Abp1p particles during actin patch assembly in buds from WT (BY4741), *sec2-41* (CBY4710), and *sec4-8* (CBY4711) cells incubated at 37°C for 60 min. **B.** Scatterplots quantifying increased average lifetime of individual Sla1p-RFP and GFP-Abp1p particles in *sec4-8* and *sec2-41* cells relative to WT, with modest lifetime increases in *sec6-4* (CBY4712) cells and no change detected in *msb3Δ msb4Δ* cells (CBY1981). **C.** Two representative tracings of GFP-Abp1p particles moving at the cell cortex in WT, *sec4-8*, and *sec2-41* cells at 37°C, as tracked using confocal video microscopy (green and red dots mark the first and last positions of the particles, respectively); tracings are oriented so that the bud cortex is up and the cell interior is down. Differences between positions (black dots) are 1 s and total elapsed times are shown above each tracing, and, below, the average velocities for GFP-Abp1p particles are plotted in the bar graph (*n* > 50 tracings). **D.** Scatterplots show total numbers of GFP-Abp1p and Sla1p-RFP particles in buds of *sec6-4*, *sec4-8*, and *sec2-41* cells relative to WT after incubation at 37°C, and in buds of *msb3Δ msb4Δ* and WT cells (*n* > 20 buds). **E.** Representative actin patch internalization defects observed in *sec4-8*, *sec2-41*, and *sla2Δ* (CBY4863) cells as shown by Abp1p-GFP “comet tails” (inserts), compared to Abp1p-GFP spots in WT (bar = 2 μm). Unless stated otherwise, for all plots, statistical differences relative to congenic WT control strains are shown by single, double, and triple asterisks indicating *p* < 0.05, 0.0015, and 0.0001, respectively.

The functional significance of Sec4p co-localization with Abp1p, Sla1p, and Las17p was apparent when analyzing characteristic properties of actin-patch assembly in a *SEC4* conditional mutant. The dynamics and morphology of actin patches provide several experimental readouts for the kinetic events before and during cortical actin polymerization that ultimately result in PM internalization [26]: (i) particle lifetimes indicate proper recruitment and turn-over of actin patch components involved in endocytic site formation; (ii) as measured by particle velocity, the rapid motility of Sla1p and Abp1p provides a readout for active actin polymerization during initial membrane invagination and inward membrane internalization, respectively; (iii) the density of actin patches is also a measure of proper subunit recruitment and stability of the internalization machinery; (iv) Abp1p accumulation on “actin comets” also indicates morphological defects in cortical actin organization. At 23°C, polarized exocytosis is unaffected in *sec4-8*^ts^ cells and is comparable to wild type [29]. However, after incubation at 37°C for 60 min, the *sec4-8*^ts^ mutation disrupts polarized exocytosis [29]. Using the motility and lifetime of actin patch components as readouts, we found that after the temperature shift, the formation of endocytic sites in these cells was also affected (Fig 2, S5 Movie); measured defects in actin patch dynamics in *sec4-8*^ts^ cells matched in magnitude the defects observed in established endocytosis mutants [26]. At 37°C, Sla1p-RFP- and GFP-Abp1p-marked actin patches persisted 1.6- and 2-fold longer in *sec4-8*^ts^ cells than in wild type, respectively (Fig 2A and 2B). Even as early as 30 min after the temperature shift to 37°C, statistically significant increases in Sla1p-RFP and GFP-Abp1p lifetimes (*p* < 0.0001; *n* = 55 cells) were detectable in *sec4-8*^ts^ cells. Actin patch dynamics and the timing of subunit recruitment were also defective in *sec4-8*^ts^ cells. In *sec4-8*^ts^ cells incubated at 37°C for 60 min, the recruitment of GFP-Abp1p to nascent actin patches was delayed 3-fold after Sla1p-RFP was first detected relative to what was observed in wild-type cells (Fig 2A), and the dynamic movement of GFP-Abp1p along the PM was 44% slower (Fig 2C). Because Sec4p GTPase activation requires its GEF Sec2p, we also tested if actin patches are affected in *sec2-41*^ts^ cells. Like *sec4-8*^ts^ cells, Sla1p-RFP lifetime (Fig 2B) and GFP-Abp1p motility (Fig 2C) were also disrupted in *sec2-41*^ts^ cells at 37°C. Moreover, the overall density of actin patches in buds declined in both *SEC4* and *SEC2* mutant cells. For *sec2-41*^ts^ and *sec4-8*^ts^ cells incubated at 37°C, there was a >70% reduction in GFP-Abp1p particles and >50% fewer Sla1p-RFP particles within buds as compared to wild type (Fig 2D). These reductions in actin patch dynamics and density within buds clearly establish that Sec4p and its regulator Sec2p affect the proper formation of endocytic sites.

Similar to what is seen in actin patch mutants, the morphology of actin patches was also aberrant in *SEC4* and *SEC2* mutant cells. In *sla2Δ* cells, for instance, instead of normal punctate GFP-Abp1p-marked actin patches, “comet tails” are observed when nucleated actin protrudes into the cytoplasm [30]. Although the deletion of *SLA2* results in a greater number of larger actin comets, we detected small comets in 31% of *sec4-8*^ts^ cells and 48% of *sec2-41*^ts^ cells at 37°C, as compared to 88% of *sla2Δ* cells and 0% of wild-type cells (*n* > 70) (Fig 2E). All told, as indicated by defects in actin patch dynamics and morphology, the proper formation of endocytic sites requires Sec4p and its GEF Sec2p.

A rapid onset of actin patch assembly defects has also been reported for temperature-conditional *SEC* mutants after incubation at elevated temperatures [5,7]. To determine the specificity of *SEC4* and *SEC2* effects on actin patches, we tested Sla1p and Abp1p dynamics in another late-acting *SEC* mutant. In *sec6-4*^ts^ cells containing a conditional mutation in the Sec6p exocyst complex subunit, there was a minor 1.2-fold and 1.3-fold increase in GFP-Abp1p and Sla1p-RFP particle lifetimes, respectively, relative to wild type after incubation at 37°C for 60 min (Fig 2B). Although Sec2p affects Sec4p activity in endocytosis, other regulators of Sec4p affect its role during exocytosis but not endocytosis. Several genes encode Sec4p GTPase-Activating Proteins (GAPs) including *MSB3* and *MSB4*, and the combined deletions of *MSB3* and *MSB4* disrupt polarized exocytosis by affecting the GTP/GDP-cycle of Sec4p [31]. However, actin patch assembly was unaffected in *msb3Δ msb4Δ* cells given that GFP-Abp1p motility and Sla1p-RFP/GFP-Abp1p lifetimes were relatively normal (though patch depolarization was observed [see below]) (Fig 2B–2D). These findings suggested that among genes required for polarized exocytosis, *SEC4* and *SEC2* have a specific role in actin patch assembly.

### Sec4p Interacts In Vitro and In Vivo with Actin Patch Subunits

**Fig 3:**
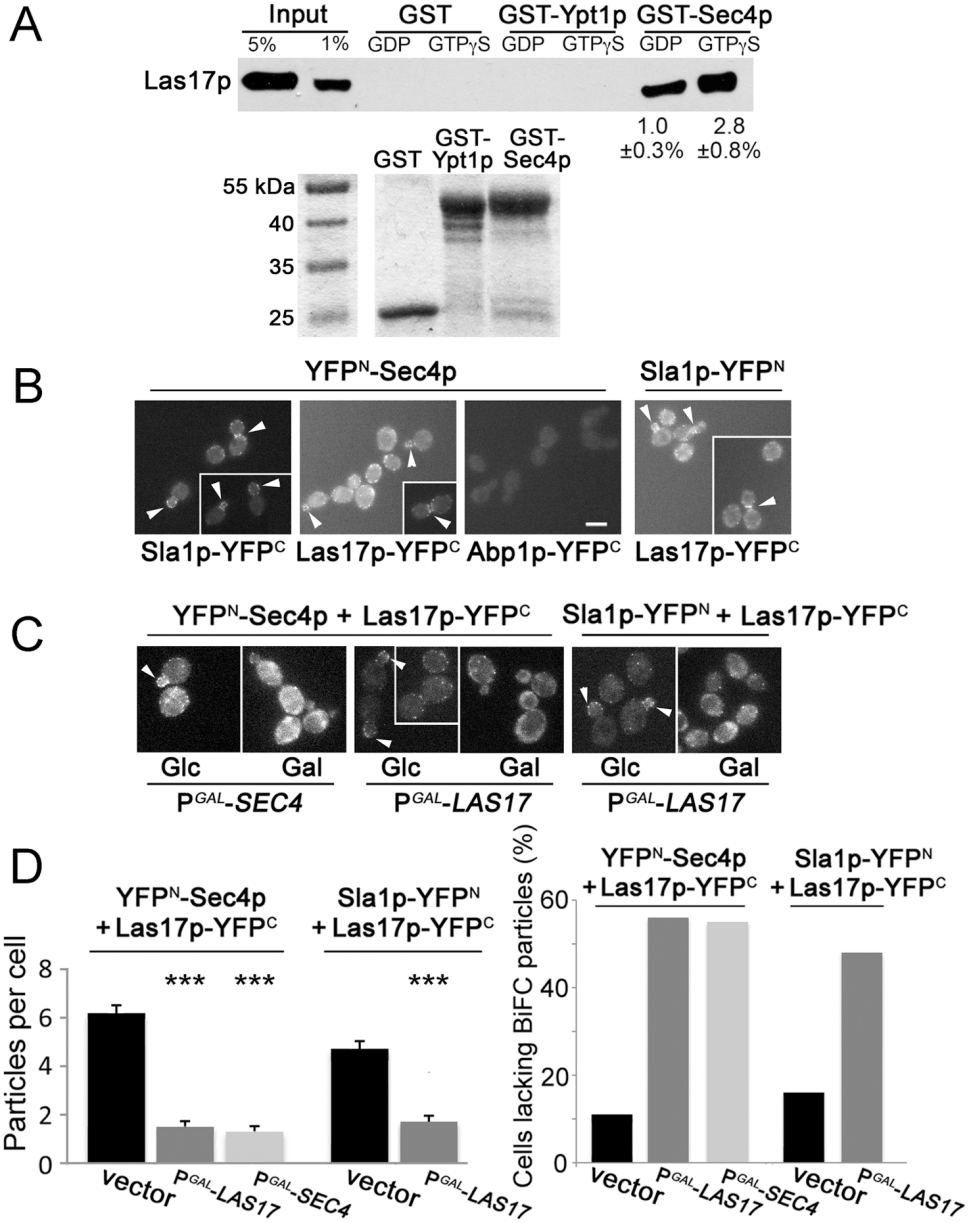
Physical interaction of Sec4p with actin patch subunits. **A.** Top panel: representative in vitro binding assay showing in vitro transcribed and translated ^35^S-Las17p binding to bacterially expressed GST-Sec4p purified and immobilized on beads prior to SDS-PAGE and autoradiography. Average percentage of input Las17p interacting with GDP- or GTPγS-bound GST-Sec4p as shown (*n* = 3). Bottom panel: SDS-PAG showing equal amounts (1 μg) of GST-Sec4p, GST, and GST-Ypt1p preloaded with GDP or GTPγS prior to ^35^S-Las17p addition. **B.** BiFC assays for cells expressing Sla2p-YFP^N^ (CBY4625) or YFP^N^-Sec4p (CBY4629) when mated with cells expressing Las17p-YFP^C^ (CBY4660), Sla2p-YFP^C^ (CBY4661), or Abp1p-YFP^C^ (CBY4632). Fluorescence at the cell cortex (arrowheads) indicates in vivo interactions at actin patches (bar = 5 μm). **C.** Competition of BiFC binding following overnight P^*GAL*^-*LAS17* induction or 6 h P^*GAL*^-*SEC4* induction with galactose (Gal), compared to no induction in glucose (Glc) medium, in cells expressing YFP^N^-Sec4p and Las17p-YFP^C^ (CBY4638). In all cells observed (including controls), non-specific cytoplasmic fluorescence increased after transfer to galactose-containing medium. **D.** Bar graphs quantifying reductions in BiFC particles within cells corresponding to images shown in panels **B** and **C** (*n* > 100 cells).

The close temporal and spatial relationship between Sec4p and actin patch subunits, Las17p in particular, suggested a possible direct physical interaction. Indeed, radiolabeled Las17p synthesized in a cell-free transcription/translation system directly interacted in vitro with GTPγS-bound glutathione S-transferase (GST)-Sec4p, expressed and purified from bacteria (Fig 3A). GST-Sec4p binding to Las17p was enhanced 2.8-fold in the presence of GTPγS as compared to GDP. In negative control assays, Las17p did not bind to GST or GST-Ypt1p whether or not GTPγS or GDP was added. Because Ypt1p is an ER/Golgi-localized small GTPase and a close Sec4p sequence homolog, we conclude that the interaction between Las17p and Sec4p represents a specific association. Using the same binding conditions, we failed to detect an interaction between bacterially expressed GST-Sec4p and in vitro translated Sla1p. These findings indicated that Sec4p specifically and directly interacts with Las17p in vitro.

To determine if the Sec4p/Las17p in vitro binding is relevant in vivo, Sec4p interactions with actin patch subunits were visualized in living cells by bimolecular fluorescence complementation (BiFC) assays [32,33] (Fig 3B–3D). In BiFC, putative interaction partners are fused with non-functional halves of a fluorescent protein (i.e., Yellow fluorescent protein [YFP]-Venus), and if they associate in a complex within ~50 Å of each other, the fused YFP^N^ amino- and YFP^C^ carboxy- halves reconstitute an intact fluorescent protein [32]. Consistent with previous findings [22], co-expression of Las17p-YFP^N^ and Sla1p-YFP^C^ generated fluorescence at cortical actin patches in all cells counted (*n* = 100), indicative of a Las17p/Sla1p interaction (Fig 3B). This same fluorescent distribution was seen in all cells (*n* = 100) when YFP^N^-Sec4p was co-expressed with Las17p-YFP^C^ or Sla1p-YFP^C^ (Fig 3B and 3D). None of the fusion proteins generated fluorescence when expressed alone. Because YFP^N^-Sec4p co-expression in cells with Abp1p-YFP^C^ also produced no fluorescence (*n* = 100 cells), we conclude that Sec4p does not interact with just any actin patch subunit, even if they briefly co-localize (Fig 3B and 3D). The YFP^N^-Sec4p BiFC interaction with Las17p-YFP^C^ was competed away by increased expression of wild-type Las17p or Sec4p (Fig 3C and 3D). P^*GAL*^-*LAS17* or -*SEC4* overexpression reduced the number of YFP^N^-Sec4p/Las17p-YFP^C^ particles, comparable to the competition observed of Las17p-YFP^N^/Sla1p-YFP^C^ BiFC interaction upon P^*GAL*^-*LAS17* overexpression (Fig 3C and 3D). As confirmed by BiFC assays, both in vivo and in vitro binding experiments indicated that Sec4p associates with the actin-patch coat complex by a direct interaction with Las17p.

### Actin Patch Subunits Have Reciprocal Effects on Sec4p and Polarized Exocytosis

**Fig 4:**
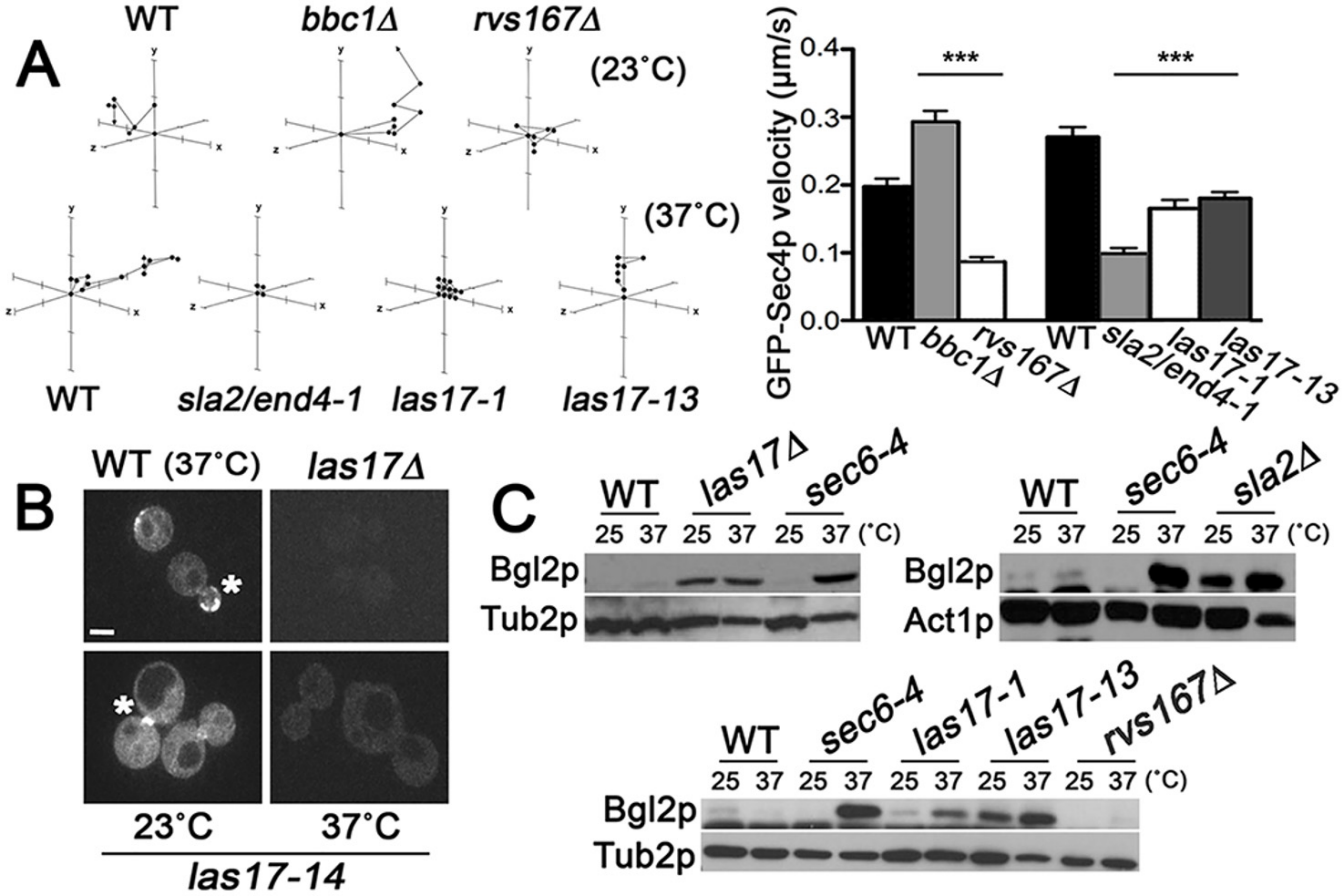
Reciprocal effects of endocytosis on polarized exocytosis. Sec4p motility is dependent on actin patch assembly. **A.** Representative tracings from three-dimensional time-lapse confocal microscopy showing GFP-Sec4p movement after its transport into photobleached zones at the bud cortex in *las17-1*^*ts*^ (CBY4356), *las17-13*^*ts*^ (CBY4357), *rvs167Δ* (CBY4733), *bbc1Δ* (CBY4373), and *sla2/end4-1*^*ts*^ (CBY4452) endocytosis-defective cells, relative to WT (CBY4741). Temperature-conditional mutations were incubated at 37°C for 2 h, whereas motility in deletion mutants was assessed at 23°C. On each axis, 0.5 μm intervals are indicated. The bar graph quantifies GFP-Sec4p particle motility at the PM for each strain (*n* > 30 particles). **B.** Images of GFP-Sec4p localization at sites of polarized growth (asterisk) in WT (BY4741), *las17Δ* (CBY1024), and *las17-14* (CBY4358) cells. Under these conditions, GFP-Sec4p was not detected on any membrane in *las17Δ* cells grown at 30°C (as shown) or in *las17-14* cells incubated at 37°C for 2 h (bar = 2 μm). **C.** Immunoblots assaying Bgl2p polarized exocytosis showing defective Bgl2p internalization in *sla2Δ* (DDY1980), *las17Δ* (DDY1709), *las17-13* (CBY4357), and *las17-1* (CBY4356) endocytosis mutants, compared to the *sec6-4* exocytosis-defective control (NY17) and congenic WT strains (BY4741 and DDY130). Bgl2p exocytosis was not defective in *rvs167Δ* cells (CBY4372). The same blots were probed for tubulin (Tub2p) or actin (Act1p) as internal loading controls.

If Sec4p regulates exo- and endocytosis in a coupled cycle, then we predicted that mutations disrupting endocytosis would have a corresponding impact on Sec4p dynamics, activity, and/or expression. Having established that *SEC4* mutations affect Sla1p and Abp1p dynamics, we tested the reciprocal, namely, whether GFP-Sec4p motility is affected by actin patch defects. In wild-type cells, the vectorial path of GFP-Sec4p changes when it arrives in the bud, making brief translational movements along the PM before disappearing [9,11]. We conducted photobleaching experiments on medium- and large-sized buds in endocytosis mutants to track individual GFP-Sec4p particles by three-dimensional time-lapse (4D) confocal microscopy (Fig 4). Specifically, we examined mutants that disrupted *BBC1* (myosin-interacting SH3 domain protein), *LAS17*, *RVS167* (amphiphysin homologue), or *SLA2* [23]. In general, we found an inverse correlation between GFP-Sec4p particle lifetimes and particle motility in these mutants. For most of these mutants, GFP-Sec4p particles persisted longer but remained static. In both *rvs167Δ* cells and *sla2*/*end4-1*^ts^ cells, GFP-Sec4p particle velocity was reduced by more than half as compared to wild type, whereas the velocity of GFP-Sec4p actually increased 67% in *bbc1Δ* cells (Fig 4A), which is consistent with the inhibitory role of Bbc1p on Las17p-dependent actin polymerization [25]. At 37°C, moderate velocity decreases were also evident in *las17-1*^ts^ and *-13*^ts^ cells (Fig 4A). To our surprise, in *las17Δ* cells grown at 30°C or in *las17-14*^ts^ cells shifted to 37°C for 60 min, GFP-Sec4p membrane localization was completely disrupted (Fig 4B). In *las17-1*, *las17-13*, and *sla2/end4-1*^ts^ cells, GFP-Sec4p particle lifetime increased by >50%, whereas it remained unchanged in *rvs167Δ* cells and decreased by 53% in *bbc1Δ* cells. As an additional assay to test how polarized exocytosis and endocytosis are inter-connected, Bgl2p secretion was analyzed in endocytosis-defective cells (Fig 4C). Bgl2p serves as a marker for polarized exocytosis because its vesicular transport is targeted to sites of polarized growth from where it is secreted out of the cell [29]. As shown for *sec6-4*^ts^ cells grown at 37°C, Bgl2p accumulated internally when polarized exocytosis is blocked (Fig 4C). Likewise, Bgl2p exocytosis was inhibited in *las17Δ*, *las17-13*, and *sla2Δ* endocytosis mutants. Because the endocytosis defect in *rvs167Δ* cells is relatively weak [34], we predicted that any coupled defect in exocytosis would also be weak. Consistent with this prediction, no Bgl2p secretion defect was detected in *rvs167Δ* cells (Fig 4C). Overall, these results support the conclusion that polarized exocytosis and endocytosis constitute a coupled transport cycle in budding yeast.

### Sec4p Polarization Affects Actin Patch Polarization

**Fig 5:**
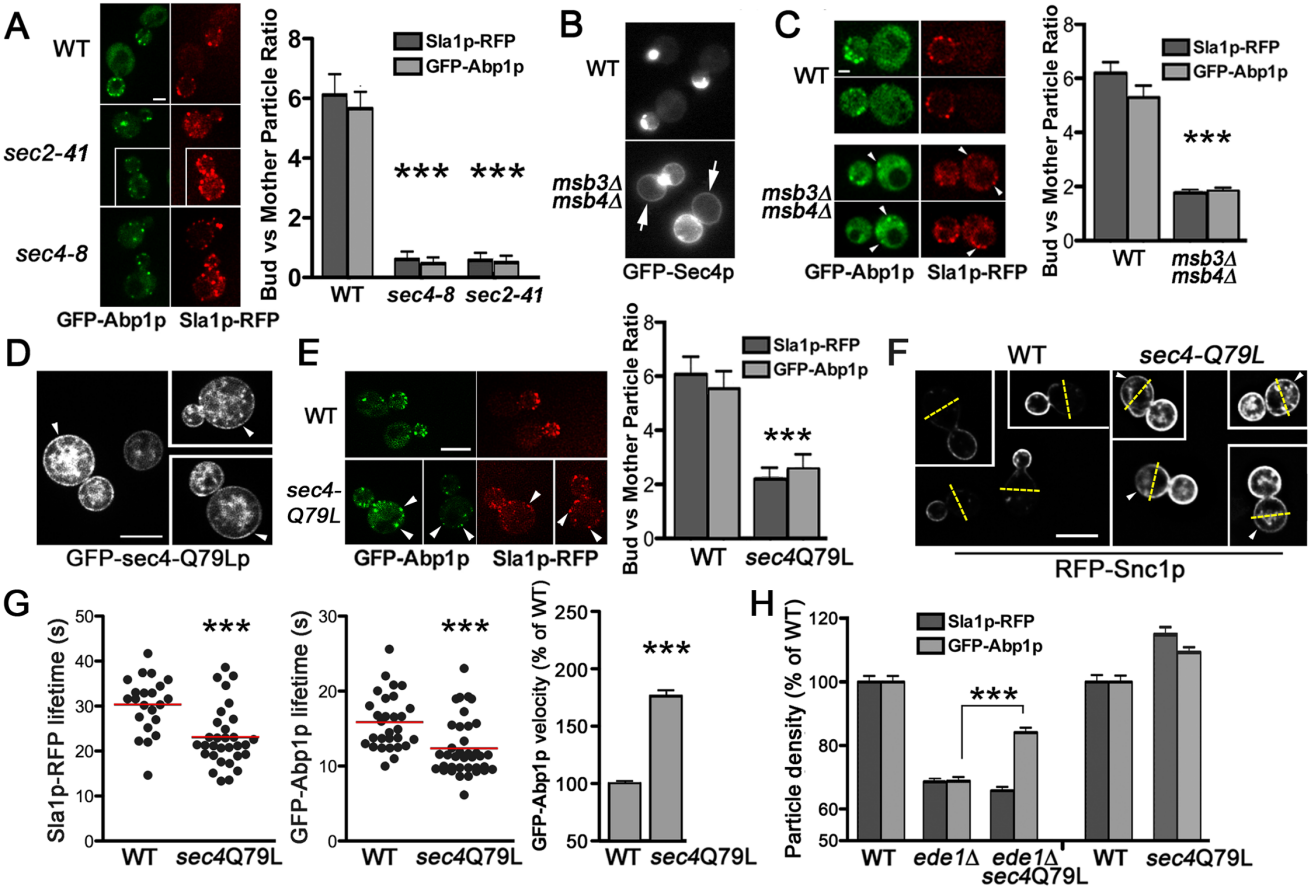
Actin patch polarization is affected by Sec4p polarization. **A.** Deconvolved medial images of Sla1p-RFP and GFP-Abp1p in WT (BY4741), *sec4-8*^ts^ (CBY4711), and *sec2-41*^ts^ (CBY4710) cells incubated at 37°C for 60 min as acquired by confocal microscopy (bar = 2 μm). Ratios of Sla1p-RFP and GFP-Abp1p localization in buds versus mother cells for WT, *sec4-8*^ts^, and *sec2-41*^ts^ cells are quantified in the bar graph (*n* > 50). **B.** Images of GFP-Sec4p fluorescence in WT (BY4741) and the corresponding depolarized GFP-Sec4p in *msb3Δ msb4Δ* (CBY1980) cells. GFP-Sec4p is dispersed around the bud and mother cell cortex (arrows) in 78% of *msb3Δ msb4Δ* cells compared to 0% in WT (*n* > 100). **C.** Images from a widefield fluorescence microscope showing Sla1p-RFP and GFP-Abp1p expressed in *msb3Δ msb4Δ* cells showing actin patch depolarization within mother cells (arrowheads), as compared to wild type. The bar graph quantifies the ratio of Sla1p-RFP and GFP-Abp1p particle localization in buds versus mother cells for WT and *msb3Δ msb4Δ* cells (*n* > 20). **D.** Deconvolved widefield microscopy images of WT (BY4741) cells expressing GFP-sec4-Q79Lp (bar = 5 μm). Arrowheads indicate depolarized distribution of GFP-sec4-Q79Lp at the PM in mother cells. **E.** Deconvolved medial images of Sla1p-RFP and GFP-Abp1p in WT (BY4741) and *sec4-Q79L* (CBY4793) cells as acquired by confocal microscopy (bar = 2 μm). Ratios of Sla1p-RFP and GFP-Abp1p localization in buds versus mother cells for WT and *sec4-Q79L* cells are quantified in the bar graph (*n* = 31). **F.** Depolarization of RFP-Snc1p cargo recycling in *sec4-Q79L* cells as compared to WT, as visualized by deconvolved widefield microscopy (bar = 5 μm). Superimposed dashed yellow lines are drawn through the middle of mother cells and arrowheads indicate PM fluorescence in the back half of mother cells furthest from the bud. **G.** Scatterplots showing lifetimes for Sla1p-RFP and GFP-Abp1p particles in WT and *sec4-Q79L* cells incubated at 23°C and, as quantified in the bar graph (right) (*n* > 30), GFP-Abp1p particle velocity increases in *sec4-Q79L* (JGY73) cells relative to its congenic WT control (KEF473A) (*n* > 70 particles). **H.** Bar graph showing Sla1p-RFP and GFP-Abp1p particle density per μm of cell surface in *ede1Δ* (CBY4775), *ede1Δ sec4-Q79L* (CBY4846), and *sec4-Q79L* (CBY4793) cells cultured at 23°C as a percentage of the particle density in WT (BY4741) cells (*n* > 50 medium- and large-budded cells). Triple asterisks (as defined in Fig 1) denote a statistically significant density increase in *ede1Δ sec4-Q79L* cells as compared to *ede1Δ* cells.

Actin patches are predominantly associated with PM regions surrounding sites of polarized growth [35]. The proximity of actin patches to these sites helps maintain the polarized distribution of proteins in buds by recycling them before they diffuse throughout the membrane into the mother cell [19,21]. In *sec2-41*^ts^ and *sec4-8*^ts^ cells at 37°C, the density of actin patches within buds sharply declined (Fig 2D) but, as observed by confocal microscopy, the total number of Sla1p- and Abp1p-RFP particles in both mother cells and their buds was equivalent to that in wild-type cells (an average of 8.4–9.9 particles/cell; *n* = 50). To determine if this discrepancy reflects a change in actin patch polarization, we analyzed the ratio of actin patches within buds relative to those within mother cells in *SEC4* and *SEC2* mutants. In *sec4-8*^ts^ and *sec2-41*^ts^ cells incubated at 37°C for 60 min, the bud-to-mother ratio of Sla1p-RFP and GFP-Abp1p was at least 10-fold less than in wild-type cells, indicating a significant loss in actin patch polarization (Fig 5A). If Sec4p polarization promotes the polarization of endocytic sites, we predicted that distributing Sec4p more uniformly around the PM would have the opposite effect and cause actin patch depolarization. As previously described, *msb3Δ msb4Δ* mutations affect Sec4p activity during exocytosis, but combined they have modest effects on actin patch assembly and motility (Fig 2C–2E). Nonetheless, we observed in *msb3Δ msb4Δ* cells that GFP-Sec4p localization spread beyond the bud and was distributed more evenly along the mother cell PM (Fig 5B). We exploited this finding to determine how Sec4p depolarization affected actin patch polarization. In these cells, Sec4p depolarization correlated with a loss in Sla1p-RFP and GFP-Abp1p asymmetry where there was a ~3-fold reduction in the normal cluster of particles in buds versus mother cells (Fig 5C). Because Msb3/4p are GAPs for some Rab GTPases other than Sec4p [36,37], we tested if Sec4p depolarization by another mechanism would also result in actin patch depolarization. The *sec4-Q79L* mutation mimics a Ras oncogenic mutation that lowers intrinsic GTPase activity and shifts more of the protein into the activated GTP-bound form, similar to what is predicted when Sec4p GAPs are deleted [38]. It is important to note that the *sec4-Q79L* mutation only affects exocytosis if cells are incubated for prolonged periods (24 h) at 14°C [36]. Despite its modest effects on exocytosis, the activated *sec4-Q79L* mutation had significant effects on actin patches even at normal growth temperatures. In all cells observed (*n* = 100), GFP-sec4-Q79Lp on the PM was depolarized into the mother cell (Fig 5D), and in *sec4-Q79L* cells, a corresponding depolarization of the actin patch subunits Sla1p and Abp1p was observed (Fig 5E). To determine if the depolarization of actin patches in *sec4-Q79L* cells impacts the polarized recycling of endocytic cargo, we analyzed the distribution of RFP-Snc1p. Snc1p is an exocytic SNARE protein that is immediately recycled after polarized exocytosis via endocytosis [39]. In 93% of wild-type cells (*n* = 100), RFP-Snc1p is concentrated at the PM in small and medium buds with little or no fluorescence in mother cells (Fig 5F). In *sec4-Q79L* cells, some increase in internal RFP-Snc1p was observed, but in a majority of these cells (87%; *n* = 100) the fluorescence was depolarized, in which RFP-Snc1p was localized on the PM in mother cells on the side opposite from the bud (Fig 5F). In *msb3Δ msb4Δ* cells, internal GFP-Snc1p accumulation obscured assessment of its depolarization at the PM. Nonetheless, these findings suggested that Sec4p polarization plays a role in the polarized enrichment of actin patches and endocytic cargo within buds.

Because loss of Sec4p function disrupted actin patch assembly and polarization, we tested if constitutive Sec4p activation might enhance actin patch assembly and/or motility. At 23°C, Sla1p-RFP lifetime in *sec4-Q79L* cells was 24% less than in wild type, which was similar to reductions in Sla1p-RFP lifetime reported for clathrin and *EDE1* endocytosis mutants [26,40]. Unlike clathrin and *EDE1* mutants, however, in *sec4-Q79L* cells, GFP-Abp1p lifetime was also reduced by 35% (Fig 5G), and in the original *sec4-Q79L* strain (JGY73), the dynamic movement of GFP-Abp1p increased by 86% relative to its congenic wild-type control (Fig 5G; this effect was not observed when *sec4-Q79L* was integrated into the BY4741 wild-type strain). Thus, Sec4-Q79Lp constitutive activation appeared to accelerate actin patch formation, which was the reciprocal effect of inactivating Sec4p (i.e., Fig 2A–2C). As predicted, because Abp1p is recruited after Sec4p, *SEC4* mutations had a slightly greater downstream impact on Abp1p lifetime as compared to Sla1p (Figs 2B, 5G and scatterplot). The effects of the constitutively active *sec4-Q79L* mutation on actin patches, and the fact that GTPγS enhanced Sec4p binding to Las17p in vitro (Fig 3A), suggested that GTP binding by Sec4p promotes actin patch assembly through Las17p.

If Sec4p interacts in vivo with Las17p and Sla1p during the slow coat assembly phase of actin patch formation, then Sec4p would act downstream after the recruitment of the early coat protein Ede1p [41,42]. Based on this pathway order, we predict that the accelerated formation of actin patches caused by the *sec4-Q79L* mutation would be epistatic to *ede1Δ* defects. In *ede1Δ* cells, the decrease in actin patch density and motility are moderate but comparable to the endocytic defects in clathrin mutants [25,26,41,42]. We found, however, that the reduction in GFP-Abp1p particles in *ede1Δ* cells was in fact partially suppressed by *sec4-Q79L* expression (Fig 5H). These experiments suggested *SEC4* stimulates the actin patch assembly pathway downstream of *EDE1*, consistent with our other findings.

### Sec4p Overrides Sla1p Inhibition of Las17p-Dependent Actin Polymerization in Vitro

**Fig 6:**
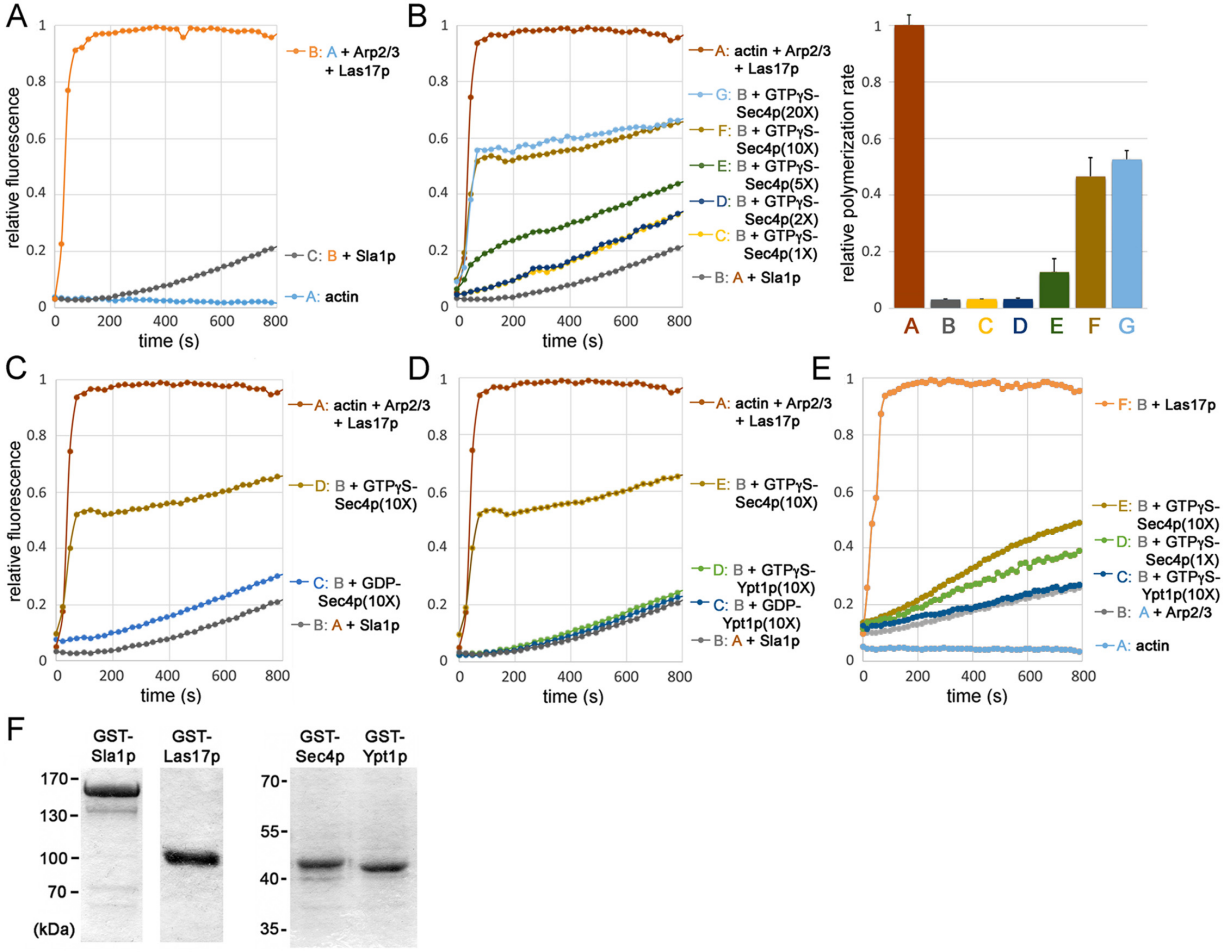
GTPγS-Sec4p overrides Sla1p inhibition of Las17p-dependent actin nucleation in vitro. All assays contained 1.5 μM actin (99% pyrene-labeled) polymerized at 30°C in the presence 75 nM Arp2/3 complex. **A.** Actin polymerization was induced upon addition of 75 nM bacterially expressed GST-Las17p, but this activation was inhibited by the addition of 75 nM full-length Sla1p (expression and purified as a GST fusion protein). **B.** Time-course (left) showing concentration-dependent effects of GTPγS-Sec4p (fused to GST) on Sla1p inhibition of Las17p, in which a 1, 2, 5, 10, or 20 X molar excess of GTPγS-Sec4p was added (relative to Las17p, Arp2/3, and Sla1p) into each actin polymerization reaction. Based on the accompanying graph, the bar graph (right) shows calculated rates of actin polymerization with increasing concentrations of GTPγS-Sec4p (rates were calculated from slopes of the linear segment of curves corresponding to half maximal polymerization). **C.** GTPγS versus GDP nucleotide dependence of Sec4p for activating actin polymerization in the presence of Sla1p and Las17p (Sec4p was added in 10 X molar excess as indicated). **D.** In contrast to the effect of GTPγS-Sec4p, GTPγS- or GDP-bound Ypt1p fails to counteract Sla1p inhibition of Las17p (Sec4p and Ypt1p were added in 10 X molar excess). **E.** In the absence of Las17p, GTPγS-Sec4p had negligible actin nucleation activity (as indicated, Sec4p was added in 1 and 10 X molar excess, and 10 X molar excess of Ypt1p was added). **F.** Affinity-purified GST fusion proteins (1 μg) used in actin polymerization assays, separated by SDS-PAGE and stained with Coomassie. All plots shown represent averages of 6–10 independent trials.

Given that Sec4p is recruited at the same time as Sla1p and Las17p during endocytic site formation, we tested whether Sec4p is also involved in the important step of Arp2/3-dependent actin nucleation, which is activated by Las17p [18]. During these time-course assays, the assembly of actin filaments involves three distinct phases [43,44]: (i) an initial nucleation lag phase, in which monomeric G-actin associates as trimers and, in conjunction with the Arp2/3 complex, forms a stable core for the addition of more actin subunits (this phase is nearly instantaneous when Las17p is added [Fig 6A]); (ii) an elongation growth phase, as denoted by the middle linear region of the sigmoidal curves, during which actin monomers are added primarily to the plus-end of growing filaments; and (iii) the final equilibrium or steady-state phase, when addition and dissociation of monomers from the respective ends of the actin filament reach a balance. To compare effects of adding additional regulatory components into these assembly reactions, polymerization rates can be calculated from the slopes of the linear regions of each plot during the filament growth phase. Both in vitro and in vivo, Sla1p inhibits Las17p nucleation activity, serving as a “checkpoint” during actin patch assembly prior to actin polymerization and membrane internalization [24,25]. To test if Sec4p affects this Sla1p—Las17p regulatory event, we performed in vitro pyrene actin polymerization assays at the physiological temperature of 30°C (Fig 6A–6E). As previously reported [24], we found that GST-Las17p stimulates Arp2/3-dependent actin polymerization, but this activity was inhibited when GST-Sla1p was added to the assay (Fig 6A). However, in a concentration-dependent manner, addition of GTPγS-GST-Sec4p counteracted Sla1p inhibition of Las17p (Fig 6B). In the presence of Sla1p, GST-Sec4p incubated with GTPγS restored actin polymerization to roughly half the maximal rate measured for assays with Las17p and Arp2/3 complex without Sla1p (Fig 6B). Although GTPγS-GST-Sec4p significantly increased the rate of actin polymerization in the presence of Sla1p, polymerization did not reach the same maximum as in assays with Las17p and Arp2/3 alone (Fig 6B). Sec4p activity in these assays was nucleotide specific given that only GTPγS-GST-Sec4p stimulated polymerization, whereas GDP-bound GST-Sec4p had no effect on Sla1p inhibition of Las17p (Fig 6C). The ability of GTPγS-Sec4p to override Sla1p inhibition was also GTPase specific because GST-Ypt1p had no effect on Sla1p-Las17p regulation of actin polymerization whether GDP or GTPγS was added (Fig 6D). In the absence of Las17p, GTPγS-Sec4p had only minimal effect on the rate of actin polymerization with added Arp2/3 complex (Fig 6E), indicating that Sec4p has no inherent actin nucleation activity and only acts as a regulator of Las17p. These data give further support to the conclusion that Sec4p directly interacts with the endocytic machinery to override a rate-limiting step in actin patch polymerization.

## Discussion

**Fig 7:**
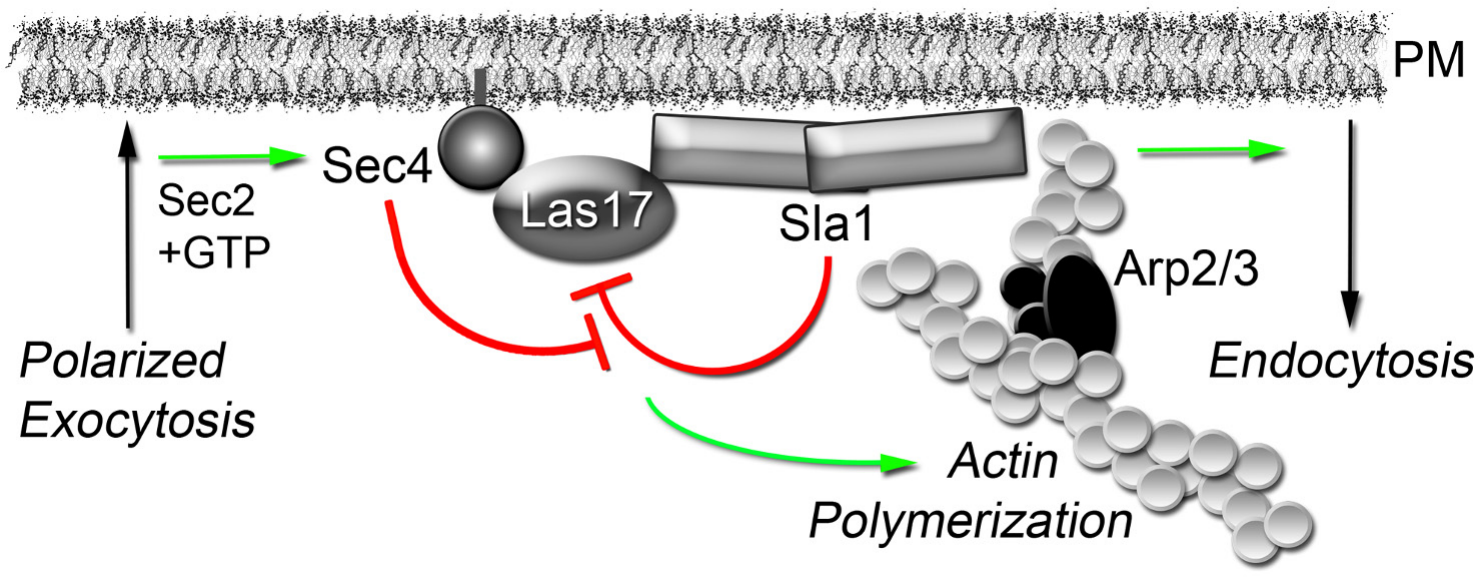
Sec4p overrides the inhibition of cortical actin polymerization to induce compensatory endocytosis. Our results supported the model in which, after the completion of polarized exocytosis, activated GTP-Sec4p deposited on the plasma membrane (PM) physically interacts with Las17p. GTP-bound Sec4p then overrides Sla1p inhibition of Las17p to promote both Arp2/3-dependent actin polymerization and the ensuing steps of endocytic membrane internalization. In the immediate vicinity around polarized sites where exocytic vesicles fuse with the PM, Sec4p directly integrates membrane transport to and from the cell surface by inducing compensatory endocytosis. In this manner, GTP-bound Sec4p promotes cortical actin polymerization after coat and adaptor proteins have initiated the formation of nascent endocytic sites.

In addition to its role in exocytosis, the Sec4p Rab GTPase also fulfills criteria expected of a direct regulator of actin patch assembly and endocytic internalization. Analysis of individual GFP-Sec4p particles in living cells after their arrival at the cell cortex indicated that Sec4p shares a brief but specific spatial and temporal co-localization with the actin patch subunits Sla1p, Las17p, and Abp1p. Akin to mutations in known actin patch components, conditional mutations in both *SEC4* and its activator *SEC2* disrupted actin patch assembly, dynamics, structure, and polarization. As shown by both in vitro and in vivo binding assays, Sec4p directly interacts with Las17p and associates in a complex with Sla1p/Las17p at nascent actin patches. Sec4p polarization was also an important determinant for polarized actin patch assembly in budding cells. Most importantly, in vitro pyrene actin polymerization assays revealed the mechanism by which Sec4p regulates actin patch formation. By overriding Sla1p inhibition of Las17p, activated Sec4p promotes actin nucleation and thereby drives forward a key regulatory step in endocytic internalization (Fig 7).

The rate-limiting step in actin filament assembly is nucleation, which involves the Arp2/3 complex and nucleation-promoting factors, the most robust being Las17p [25,45]. As a clathrin adaptor, Sla1p is required for the endocytic internalization of membrane protein cargo. However, Sla1p and other inhibitors also restrain Las17p activity and thereby regulate the final burst of cortical actin polymerization, which ultimately provides the force needed for membrane internalization [24,25,46]. Sec4p arrives at nascent endocytic sites during this brief period, and Sec4p relieved the inhibition of Las17p nucleation by Sla1p in vitro. Sec4p largely restores actin polymerization rates but, even at saturating concentrations, Sec4p did not completely reverse Sla1p inhibitory effects. Under these assay conditions, the relative maximum level of actin polymerization was not attained, suggesting that the monomer—polymer equilibrium was affected, perhaps due to an increased rate of filament depolymerization or treadmilling. Sla1p is also not the only inhibitor to keep Las17p in an inactive state. Although Syp1p binds to Sla1p, Syp1p also inhibits Las17p activity, possibly in a Sla1p-independent manner [24,25,46]. Nonetheless, in our in vitro assays, Sec4p counteracted Sla1p inhibition without Syp1p, suggesting that Sec4p inhibition of Sla1p does not involve Syp1p. Because Sec4p directly binds Las17p, Sec4p might directly interfere or compete with the physical interaction between Sla1p and Las17p. Although in vitro-activated Sec4p was required in molar excess to effectively override Sla1p inhibition, the effective concentration of Sec4p in vivo is likely to be significantly greater given that Sec4p particles are concentrated on the membrane. We note that bacterially expressed Sec4p is not posttranslationally lipidated, which might also reduce its activity in these assays. Previous in vitro reconstitution of actin assembly from yeast extracts did not identify Sec4p as a contributing factor in Las17p-dependent actin nucleation [47]. However, this biochemical approach would have excluded membranes and membrane-bound regulators such as Sec4p. Apart from Las17p-dependent nucleation of cortical actin, other activators also contribute to the initial seeding events for actin polymerization. In fission yeast, it is proposed that cofilin-severed actin filaments bind specific adapter proteins at nascent sites of endocytosis to provide the initial filaments from which the Arp2/3 branched actin network grows [48]. We conclude that Sec4p plays an important role in the Sla1p/Las17p regulation of Arp2/3-dependent actin polymerization, but this mechanism likely works in parallel with other regulators that independently promote cortical actin formation.

The initial formation of nascent endocytic sites appears to be largely dictated by cargo—adapter interactions [18], but downstream events in actin patch assembly also regulate the rate of endocytosis [7,24,49–52]. At the PM, the most rapidly maturing actin patches coincide with the immediate regions around newly delivered exocytic vesicles [20]. One interpretation of this result is that the concentration of endocytic cargo at these regions triggers adaptor packaging and endocytic site formation. Although this proposal is consistent with computational modeling, it has been noted that major cargo-binding adaptors arrive later in the process of actin patch assembly [18]. If general cargo delivery by exocytosis were the general cue for initiating endocytosis, then any mutation in exocytic trafficking would be predicted to disrupt endocytosis, which is not the case [5]. Alternatively, a specific regulator of exocytic vesicle trafficking could be the trigger. We propose that Sec4p is that trigger, which couples transport to and from the PM. In this way, Sec4p is the last of many GTPases in a Rab cascade [53] that sequentially regulates membrane trafficking out to the cell cortex, and back again, to ensure a directional flow of membrane traffic throughout the cell. Because Sec4p is first recruited to the secretory pathway at the Golgi, this model also explains why endocytosis is generally more affected by late *SEC* genes as compared to early *SEC* genes, which affect the exocytic pathway before the Golgi [5,8]. We also observed that the reciprocal was true; namely, that the cellular distribution of Sec4p was dependent on Las17p. The severe mis-localization of GFP-Sec4p in *las17Δ* and *las17-14* cells was particularly surprising. Although other alleles of *LAS17* affected GFP-Sec4p motility on the PM, at high temperatures, *las17-14* specifically reduced all membrane-bound GFP-Sec4p. Given that the *las17-14* allele is synthetically lethal with a deletion mutation that removes the proline-rich carboxy-terminal region of Pan1p (*pan1*ΔPRD) [54], the specific effect of *las17-14* on Sec4p localization might involve Pan1p and the broader Las17p regulatory network. Pan1p interacts in an endocytic scaffolding/signaling complex with End3p and Sla1p [55], which together might modulate Sec4p activity and localization. Alternatively, Las17p binding contributes to Sec4p stability, and this physical interaction is abolished in *las17Δ* or *las17-14* cells but not in *las17-13* or *las17-1* cells. Based on our results, Sec4p is a bifunctional regulator of both polarized exocytosis and endocytosis.

A convergence of exocytosis and endocytosis has also been described in filamentous fungi, in which the “Spitzenkörper” serves as a recycling compartment at the apical tip of hyphae that maintains polarized growth [56–58]. This specialized compartment contains recycled PM proteins, polarisome components, and exocyst subunits as well as Sec4p. In *S*. *cerevisiae*, a Spitzenkörper-like membrane structure containing both Sec4p and the Cdc42p repressor Bem3p has been reported [15]. In addition to Sec4p and Bem3p, this compartment contains both endocytic and exocytic markers and is only observed early during bud emergence at polarized tips, close to but not directly associated with the PM. In contrast, our measurements were restricted to Sec4p particles on the PM and only involved later-staged medium/large-budded cells. Based on these facts, the Sec4p-actin patch interactions we analyzed are independent of any Spitzenkörper-related recycling compartment. Nonetheless, Sec4p polarization did have significant impact on the polarization of nascent endocytic sites on the PM. Apart from the general inhibition of actin patch assembly, actin patch enrichment within the bud became depolarized following Sec4p inactivation. The depolarization of Sec4p itself, as observed in *msb3Δ msb4Δ* cells, also correlated with depolarization of actin patches even though patch assembly was only mildly affected. In addition, the depolarized localization of activated sec4-Q79Lp correlated with both actin patch depolarization and defects in the polarized recycling of endocytic membrane cargo. Of course, other mechanisms also exist to promote actin patch polarization, including the effects of distinct lipid subdomains within the PM of budding cells as well as polarization processes acting downstream of Cdc42p [18,59]. However, our data suggest that polarized exocytosis deposits Sec4p at sites on the PM where it can stimulate cortical actin polymerization nearby. By internalizing material adjacent to sites of polarized growth before extensive lateral diffusion can occur, we propose this mechanism prevents the escape of membrane proteins from the bud and reinforces the separation between mother and daughter cells provided by the septin barrier [60,61].

In metazoans, Cdc42 plays a central role in integrating exocytosis and endocytosis, for example, in the endocytic recycling of junctional proteins [4,62]. In Xenopus eggs, Cdc42 binds N-WASp and facilitates filamentous actin assembly around vesicles docked at the PM, which reconfigures cortical granules for compensatory endocytic internalization after exocytosis [2]. Unlike metazoan N-WASp, however, the yeast homologue Las17p does not directly interact with Cdc42p [63], and the inter-relationship between Cdc42p and transport cycles involving polarized exocytosis and endocytosis are unclear [20]. Thus, the “kiss-and-coat” mode of compensatory endocytosis in Xenopus [64], or “kiss-and-run” recycling of presynaptic exocytic vesicles [3], does not appear to apply to budding yeast. Our results are, however, consistent with findings in *Caenorhabditis elegans*, implicating the Sec4p homologue Rab3 in coordinating the exocytosis/endocytosis cycle of synaptic vesicles [65]. We therefore propose by this mechanism of compensatory endocytosis that Sec4p homologues in other polarized cell types might augment or supplant Cdc42p in maintaining a balance of membrane going to and from the PM.

## Materials and Methods

### Strains and Plasmids

Yeast strains used are listed in the S1 Table and plasmids in S2 Table. All fusions were functional as tested by complementation of corresponding mutant defects.

### Fluorescence Microscopy

Widefield fluorescence microscopy was performed as previously described [66]. Confocal images were captured on a Zeiss Axio Observer.Z1 microscope (Carl Zeiss International, Oberkochen, Germany) equipped with a CSU-10 Nipkow spinning disc (Yokogawa Electronic Corp., Tokyo, Japan), and Z-stacks were acquired using an Improvision Piezo Focus Drive. Z-stacks were separated by 0.5 μm and spanned, at minimum, the entire cell width. Images were acquired using a Zeiss 100 X 1.4 NA plan-apochromat oil immersion lens and a Hamamatsu EM-CCD C9100-13 camera (Hamamatsu Photonics, Hamamatsu, Japan) mounted on a 1.5 X C-mount, and digital analysis and deconvolution was done using Volocity software (Improvision Inc., Lexington, MA). GFP and RFP fluorophores were excited with 491 nm and 561 nm lasers, respectively; emitted light was filtered with GFP ET520/40M or RFP ET593/40M emission filters (Chroma Technology Corp., Rockingham, VT). Cells were mounted directly onto glass slides in synthetic medium, and images were acquired with equivalent exposures and laser power unless specified otherwise.

To track newly generated individual GFP-Sec4p particles after entering the daughter bud, the entire bud was first photobleached using a 300 mW solid-state 405 nm laser (Lasiris ColdRay Laser, Stockeryale Inc., Salem, NH). The FRAP laser was used at maximum power for 250 ms when analyzing GFP-Sec4p colocalization with Sla1p-RFP and Abp1p-RFP and 40 ms for photobleaching in GFP-Sec4p/Las17p-RFP-containing cells. The exposure time was 61 ms (71% laser power) and 1.0 s (67% power) for co-localization experiments involving GFP-Sec4p and Sla1p-RFP, respectively; 51 ms (65% power) and 505 ms (69% power) for GFP-Sec4p and Abp1p-RFP, respectively; and 500 ms (35% power) and 1 s (40% power) for Las17p-RFP, respectively. GFP-sec4-Q79L was imaged on the confocal microscope at 300 ms exposure at 70% power. Actin comet tails were detected by observing actin patch formation in cells over a 90 s period using widefield video microscopy. Abp1p-GFP comet tail images were captured with a 400 ms exposure at 17% arc lamp intensity and full gain. RFP-Snc1p was visualized by widefield microscopy using 50 ms exposures at 17% arc lamp intensity.

### Image Analysis

The following guidelines were used for the quantification of particle tracking and co-localization in movies and captured images: (i) for tracking reliability in movies, only new GFP-Sec4p particles transported to the bud cortex, and whose entire lifespan was captured, were analyzed; (ii) only particles in medium and large buds were analyzed because within smaller buds, particles crossed paths due to high density; (iii) for movies of all cells analyzed, fluorescent particles that crossed paths were excluded from analysis; (iv) Sec4p particles were followed immediately after photobleaching as each Sec4p particle arrived at the bud cortex and ended after the particle was no longer visible at any focal plane within the cell; (v) actin patch subunits were tracked when particles first appeared on the PM and ended when they were no longer detectable within any focal plane; (vi) in movies and still images, individual particles that could not be completely distinguished from local fluorescence background were excluded from analysis; (vii) only those particles in which fluorescence overlap persisted over three or more movie frames were counted as being co-localized; and (viii) given that slight positional changes between acquisition of each fluorescence image might cause particles to appear juxtaposed, co-localization was defined as when the majority of fluorescence pixels overlapped between two individual particles.

Particles were tracked using the manual tracking function of the visualization and quantification module in Volocity. The manual tracking function was also used for 4-D confocal microscopy analysis of particle movement. Particle tracings and velocity data were generated using the Volocity visualization and quantification module. Kymographs were generated using the multiple kymograph module of ImageJ (http://rsb.info.nih.gov/ij/). To determine the peak particle fluorescence at the PM on kymographs, Voxel Spy from Volocity was used to determine the frame with highest voxel pixel intensity from videos recording the entire lifetime of each particle; if peak voxel intensity remained constant for multiple frames, then the frame with the highest area of peak intensity was chosen. Images of actin comet tails were deconvolved by iterative restoration with a 95% confidence threshold using Volocity 3-D deconvolution based on a theoretical PSF calculated from emissions at 509 nm with a 1.40 NA. All images were equivalently processed using Photoshop (Adobe Systems, San Jose, CA). Movies were rendered and characters were added using Sony Vegas Pro 8.0 (Sony, Tokyo, Japan).

### BiFC Protein Interaction Assays

BiFC was performed as previously described [67] with modifications. For constant expression levels, YFP^N^- and YFP^C^-fusion constructs were integrated using the modified vectors pHVF1-CT, pUVF2-CT, and pHVF2-NT (gifts from Dr. Christopher Loewen, UBC). Haploid transformants expressing YFP^N^- and YFP^C^-fusions, respectively, were mated and BiFC assays were conducted by widefield fluorescence microscopy on the resulting diploid cells. For each image, a single optical section was acquired using an YFP filter as previously described [67]. Exposure times were 500 ms at full gain using an arc lamp intensity set at 55%, except for YFP^N^-Sec4p/Abp1p-YFP^C^ and Las17p-YFP^N^/Sla1p-YFP^C^ analysis, for which the intensity was set at 100%. The images were deconvolved using the Volocity 3D deconvolution theoretical point spread function and calculated based on emissions at 520 nm with a 1.40 NA.

### In Vitro Protein Binding Assays and Bgl2p Polarized Exocytosis Assay

For affinity purification, GST-Sec4p, GST-Ypt1p, and GST were expressed in BL21(DE3) RIL cells (Agilent Technologies, Santa Clara, CA) following induction with 1 mM IPTG for 14–16 h at 23°C. Cells were lysed by sonication in purification buffer (125 mM Tris pH 8.0 150 mM NaCl) and the GST-fusion proteins were immobilized on magnetic glutathione beads (Pierce Biotechnologies Inc., Rockford, IL) after overnight incubation at 4°C. Protein-bound beads were washed 3 times with purification buffer and, after Bradford analysis of protein content, 250 μg of bound protein was washed 3 times with 150 mM Tris pH 7.5, 10 mM EDTA, 250 mM NaCl, 0.5% [v/v] Triton X-100 to remove any GTP or GDP already bound by the isolated GST-Sec4p and GST-Ypt1p. Then, after 3 washes with binding buffer (150 mM Tris pH 7.5, 1.5 mM MgCl_2_, 250 mM NaCl, 0.5% [v/v] Triton X-100), beads were resuspended in binding buffer containing either 1.5 mM GDP or 1.5 mM GTPγS and incubated for 30 min at 23°C. ^35^[S]-labeled Las17p-Myc, corresponding to 10 μl of product from the TNT T7 Coupled Reticulocyte Lysate System (Promega, Madison, WI), was added to 1 μg of each immobilized GST-fusion sample and incubated for 1 h at 23°C. After 6 x 5 min washes with binding buffer containing 1.5 mM GDP or GTPγS, respectively, bound ^35^[S]-Las17p-Myc was eluted by boiling in SDS sample buffer, separated by SDS-PAGE, and gels were fixed, dried, and exposed on film. Bgl2p polarized exocytosis was assayed as previously described [66].

### In Vitro Pyrene-Actin Polymerization Assay and Affinity Purification of Fusion Proteins

For purification of GST-fusion proteins from bacterial extracts, and their subsequent use in actin polymerization assays, coding sequences for *SLA1*, *LAS17*, *SEC4*, and *YPT1* were sub-cloned into *Bam*HI and *Sal*I sites in pGEX-4T-1 (GE Healthcare Life Sciences, Pittsburg, PA). For protein expression, plasmids were transformed into the *Escherichia coli* strain BL21 (DE3) RIL. To induce GST-fusion protein expression, 250 ml of early log-phase cultures grown in LB at 37°C were treated with 1 mM IPTG and, after an additional 2 h of growth at 23°C, cells were pelleted by centrifugation at 5,000 x *g* for 30 min at 4°C. After resuspension in 20 ml binding buffer (125 mM Tris pH 8.0, 150 mM NaCl) containing EDTA-free protease inhibitors (Roche, Basel, CH), cells were lysed by sonication using a digital sonifier (Branson Ultrasonics Co., Danbury, CT) at 20% amplitude for 3 min with cycle bursts of 1 s on and 3 s off. Cell debris was pelleted by centrifugation at 25,000 x *g* for 1 h at 4°C and GST-fusion proteins were purified from the supernatant by binding overnight to glutathione magnetic beads (Pierce Biotechnology Inc., Rockford, IL) at 4°C. Beads were washed 6 times with binding buffer, and fusion proteins were eluted after a 30 min incubation in elution buffer (125 mM Tris pH 8.0, 150 mM NaCl, 50 mM glutathione) containing HALT protease inhibitor (Pierce Biotechnologies Inc. Rockford, IL) at 4°C. The elutions were repeated 4 times and eluents were pooled. Before addition to pyrene-actin polymerization assays, fusion proteins were concentrated using Amplicon size exclusion spin columns (100k for GST-Sla1p, 50K for GST-Las17p, and 35k for GST-Sec4p and GST-Ypt1p) by centrifugation at 7,000 x *g* for 30 min at 4°C. Concentrated fusion protein was diluted in G buffer for actin polymerization assays. Protein concentrations were determined by Bradford analysis (Sigma-Aldrich, St. Louis, MO).

Actin polymerization experiments were performed as previously described [20]. Reaction mixtures contained 1.5 μM depolymerized rabbit actin (99% pyrene-labeled) (Cedarlane Labs, Burlington, ON), 75 nM bovine Arp2/3 complex (Hypermol EK, Bielefeld, Germany), and 75 nM GST-Sla1 and/or GST-Las17, along with varying concentrations of GST-Sec4 and GST-Ypt1 incubated with 1.5 mM GTPγS or GDP. Reactions were initiated upon addition of 1/10 volume of polymerization buffer (500 mM KCl, 20 mM MgCl_2_, 10 mM ATP). Actin polymerization was measured at 30°C using an Infinte 200M PRO microplate reader (Tecan Mannedorf, CH) with excitation and emission wavelengths of 365 nm and 406 nm, respectively. Actin polymerization rates were calculated from the slope of the linear portion of assembly curves.

### Statistical Analysis

Statistical differences between datasets were analyzed with two-tailed unpaired Student’s *t* tests from which *p*-values were derived. Scatterplots were generated using Graphpad Prism 5.0 (Graphpad Software, La Jolla, CA) where means are indicated with vertical lines. In bar graphs, statistical data is shown as mean values with error bars representing SEM.

## Supporting Information

S1 Data Supporting data. (XLSX)

S1 Table *S*. *cerevisiae* strains. Unless otherwise referenced, all strains were created as part of this study [68–80].
(DOCX)

S2 Table Plasmids. Unless otherwise referenced, all plasmids were created as part of this study [68–80].
(DOCX)

S1 Movie Photobleaching enables single particle tracking of GFP-Sec4p at the cell cortex. Time-lapse videos of two representative wild-type cells (BY4741) expressing both GFP-Sec4p and Abp1p-RFP. Large white circles indicate the photobleached region (bud membrane), and asterisks indicate the duration of the laser burst. Green arrowheads indicate cortical GFP-Sec4p particles, RFP-Abp1p is indicated by red arrowheads, and yellow arrowheads indicate the transitional overlap of GFP-Sec4p and RFP-Abp1p fluorescence. Total acquisition time is 90 s edited and compressed to play at 5 frames/s (for technical reasons, 3 frames during laser photobleaching were excluded).
(MP4)

S2 Movie Las17p-RFP and GFP-Sec4p particles co-localize at cortical acting patches. Time-lapse video of a wild-type cell expressing RFP-Las17p and GFP-Sec4p, immediately after photobleaching. Particle co-localization at cortical actin patches is indicated by circles where red and green arrowheads indicate Las17p and cortical Sec4p, respectively, and yellow arrowheads indicate their temporal and spatial overlap. Total acquisition time is 2 min, compressed into 5 frames/s.
(MP4)

S3 Movie Sla1p-RFP and GFP-Sec4p co-localize at cortical actin patches. Time-lapse video of a representative wild-type cell expressing Sla1p and GFP-Sec4p, immediately after photobleaching. Particle co-localization at cortical actin patches is indicated by circles where red and green arrowheads indicate Sla1p and cortical Sec4p, respectively, and yellow arrowheads indicate their temporal and spatial overlap. Total acquisition time is 1 min, compressed into 4 frames/s.
(MP4)

S4 Movie GFP-Sec4p particles co-localize with Abp1p-RFP at cortical acting patches. Time-lapse video of a wild-type cell expressing GFP-Sec4p and Abp1-RFP, immediately after photobleaching. Particle co-localization at cortical actin patches is indicated by circles where green and red arrows indicate cortical GFP-Sec4p and Abp1p-RFP, respectively, and yellow arrowheads indicate their coincident overlap. Note that GFP-Sec4p precedes Abp1p-RFP at actin patches, whereas Las17p and Sla1p precede the appearance of GFP-Sec4p particles. Total acquisition time is 2 min, compressed into 5 frames/s.
(MP4)

S5 Movie *SEC4* and *SEC2* function is required for normal actin patch polarization and dynamics. WT (BY4741), *sec2-41* (CBY4710), and *sec4-8* (CBY4711) cells expressing Sla1p-RFP and Abp1-GFP after an incubation at 37°C for 60 min. Particles are tracked immediately after photobleaching where circles indicate examples of co-localization. Red arrowheads indicate Sla1p-RFP particles, green arrowheads indicate Abp1p particles, and yellow arrowheads indicate temporal and spatial overlap. Total acquisition time is 2 min compressed into 5 frames/s.
(MP4)

## Abbreviations

BiFC: bimolecular fluorescence complementation
Gal: galactose
GAPs: GTPase-Activating Proteins
GEF: guanine nucleotide exchange factor
GFP: Green fluorescent protein
Glc: glucose
GST: glutathione S-transferase
PM: plasma membrane
RFP: Red fluorescent protein
WASp: Wiskott–Aldrich Syndrome protein
YFP: Yellow fluorescent protein
WT: wild type
