# Supplementary material for: Polarized Exocytosis Induces Compensatory Endocytosis by Sec4p-Regulated Cortical Actin Polymerization
Source: PLoS Biol. 2016 Aug 15;14(8):e1002534. doi: 10.1371/journal.pbio.1002534 (PMC4985162; doi:10.1371/journal.pbio.1002534)
Supplement: S1 Table — Unless otherwise referenced, all strains were created as part of this study [68–80]. (DOCX) [file pbio.1002534.s002.docx]

**S1 Table:** ***S. cerevisiae* strains.** Unless otherwise referenced, all strains were created as part of this study.

| Strain | Genotype | Source |
| --- | --- | --- |
| BY4741 | *MAT*a *his3∆1 leu2∆0 met15∆0 ura3∆0* | [68] |
| BY4742 | *MAT*α *his3∆1 leu2∆0 lys2∆0 ura3∆0* | [68] |
| CBY31 | *MAT*a *leu2-3,122 lys2-801 ura3-52 his3∆200 trp1∆901 suc2∆9* |  |
| CBY1024 | *MAT***a** *his3∆1 leu2∆0 lys2∆0 ura3∆0* *las17∆::kan-MX4* |  |
| CBY1980 | *MAT***a** *his3∆1 leu2∆0* *met15∆0* *lys2∆0 ura3∆0* *msb3∆*::*kan-MX4 msb4∆*::*kan-MX4* |  |
| CBY1981 | *MAT***a** *his3∆1 leu2∆0* *ura3∆0* *msb3∆*::*kan-MX4 msb4∆*::*kan-MX4* |  |
| CBY4356 | BY4741 *las17-1:kan-MX4* | P. Hieter, UBC |
| CBY4357 | BY4741 *las17-13:kan-MX4* | P. Hieter, UBC |
| CBY4358 | BY4741 *las17-14:kan-MX4* | [69] |
| CBY4372 | BY4741 *rvs167∆*::*kan-MX4* | [68] |
| CBY4373 | BY4742 *bbc1∆*::*kan-MX4* | [68] |
| CBY4374 | BY4741 *sla1∆::kan-MX4* | [68] |
| CBY4621 | SEY6210 *LAS17-*YFP^N^*:HIS3* |  |
| CBY4629 | SEY6210 *HIS3:*P*^ADH1^-*YFP^N^*-SEC4* |  |
| CBY4632 | CBY31 *ABP1-*YFP^C^*:URA3* |  |
| CBY4635 | CBY31 *HIS3:*P*^ADH1^-*YFP^N^*-SEC4* |  |
| CBY4638 | *MAT*α/*MAT***a** *leu2-3,122/leu2-3,122 lys2-801/lys2-801 ura3-52/ura3-52 his3∆200/his3∆200 trp1∆901/trp1∆901 suc2∆9/suc2∆9 HIS3:*P*^ADH1^-*YFP^N^*-SEC4/SEC4 LAS17-*YFP^C^*:URA3/LAS17* |  |
| CBY4645 | *MAT*α/*MAT***a** *leu2-3,122/leu2-3,122 lys2-801/lys2-801 ura3-52/ura3-52 his3∆200/his3∆200 trp1∆901/trp1∆901 suc2∆9/suc2∆9 HIS3:*P*^ADH1^-*YFP^N^*-SEC4/SEC4 ABP1-*YFP^C^*:URA3/ABP1* |  |
| CBY4657 | SEY6210 *LAS17-*YFP^C^*:URA3* |  |
| CBY4677 | BY4741 *SLA1-*RFP*:HIS3* |  |
| CBY4679 | CBY31 *SLA1-YFP^C^:URA3* |  |
| CBY4681 | SEY6210 *SLA1-*YFP^C^*:URA3* |  |
| CBY4686 | BY4741 *ABP1-*RFP*:HIS3* |  |
| CBY4689 | BY4741 *LAS17-*RFP*:HIS3* |  |
| CBY4710 | *MAT***a** *his3∆1 leu2∆0 lys2∆0 ura3∆0* *sec2-41:kan-MX4* | C. Boone, U. Toronto |
| CBY4711 | *MAT***a** *his3∆1 leu2∆0 lys2∆0 ura3∆0* *sec4-8:kan-MX4* | C. Boone, U. Toronto |
| CBY4712 | *MAT***a** *his3∆1 leu2∆0 lys2∆0 ura3∆0* *sec6-4:kan-MX4* | C. Boone, U. Toronto |
| CBY4742 | *MAT*α/*MAT***a** *leu2-3,122/leu2-3,122 lys2-801/lys2-801 ura3-52/ura3-52 his3∆200/his3∆200 trp1∆901/trp1∆901 suc2∆9/suc2∆9 LAS17-*YFP^N^*:HIS3/LAS17 SLA1-*YFP^C^*:URA3/SLA1* |  |
| CBY4749 | *MAT*α/*MAT***a** *leu2-3,122/leu2-3,122 lys2-801/lys2-801 ura3-52/ura3-52 his3∆200/his3∆200 trp1∆901/trp1∆901 suc2∆9/suc2∆9 HIS3:*P*^ADH1^-*YFP^N^*-SEC4/SEC4 SLA1-*YFP^C^*:URA3/SLA1* |  |
| CBY4759 | *MAT***a** *his3∆1 leu2∆0 lys2∆0 ura3∆0* *sec4∆::kan-MX4* [GFP*-SEC4 URA3 CEN*] |  |
| CBY4768 | *MAT***a** *his3∆1 leu2∆0 met15∆0 ura3∆0* *sec2-41:kan-MX4* *SLA1-*RFP:*HIS3* [P*^ACT1^-*GFP*-ABP1 URA3 CEN*] |  |
| CBY4775 | BY4742 *ede1∆::kan-MX4* | [69] |
| CBY4787 | *MAT***a** *his3∆1 leu2∆0 lys2∆0 ura3∆0 sec4-Q79L:HIS3:sec4∆::kan-MX4* [P*^ACT1^-*GFP*-ABP1 URA3 CEN*] |  |
| CBY4793 | *MAT***a** *his3∆1 leu2∆0 lys2∆0 ura3∆0 sec4-Q79L:HIS3:sec4∆::kan-MX4* |  |
| CBY4805 | BY4742 *SLA1-RFP:HIS3* [P*^ACT1^-GFP-ABP1 URA3 CEN*] |  |
| CBY4810 | *MAT*α *his3∆1 leu2∆0 ura3∆0 ede1∆::kan-MX4 SLA1-*RFP*:HIS3* [P*^ACT1^-*GFP*-ABP1 URA3 CEN*] |  |
| CBY4846 | *MAT*α *his3∆1 leu2∆0 ura3∆0 ede1∆::kan-MX4 SLA1-*RFP*:HIS3 sec4-Q79L:HIS3:sec4∆::kan-MX4* [P*^ACT1^-*GFP*-ABP1 URA3 CEN*] |  |
| CBY4863 | BY4742 *sla2∆::kan-MX4* | [68] |
| DDY130 | *MAT***a** *his3∆200 leu2-3,112 lys2-801 ura3-52* | D. Drubin, UC, Berkeley |
| DDY904 | *MAT*α *his3∆200 leu2-3,112 lys2-801 ura3-52* | D. Drubin, UC, Berkeley |
| DDY1438 | *MAT*α *his3∆200 leu2-3,112 lys2-801 ura3-52 las17∆::URA3* | D. Drubin, UC, Berkeley |
| DDY1980 | *MAT***a** *his3∆200 leu2-3,112 lys2-801 ura3-52 sla2∆::URA3* | D. Drubin, UC, Berkeley |
| JGY73 | *MAT***a** *his3∆200 leu2∆1 lys2-801 trp1∆63 ura3-52 sec4-Q79L* | [27] |
| KEF473A | *MAT***a** *his3∆200 leu2∆1 lys2-801 trp1∆63 ura3-52* | [27] |
| NY17 | *MAT***a** *sec6-4^ts^ ura3-52* | [70] |
| RH286-1C | *MAT*a *leu2 ura3 his4 bar1 end4-1(ts)* | [71] |
| RSY255 | *MAT*α *ura3-52 leu2-3,112* | [72] |
| SEY2102 | *MAT*α *his4-519 leu2-3,112 ura3-52 bgl2::URA3* | [73] |
| SEY6210 | *MAT*α *ura3-52 his3∆200 lys2-801am leu2-3,112 trp1∆901 suc2∆9* | [74] |
| W303-1A | *MAT*a *leu2-3,112 ura3-1 his3-11 can1-100 ade2-1* |  |
