# Supplementary material for: Polarized Exocytosis Induces Compensatory Endocytosis by Sec4p-Regulated Cortical Actin Polymerization
Source: PLoS Biol. 2016 Aug 15;14(8):e1002534. doi: 10.1371/journal.pbio.1002534 (PMC4985162; doi:10.1371/journal.pbio.1002534)
Supplement: S2 Table — Unless otherwise referenced, all plasmids were created as part of this study [68–80]. (DOCX) [file pbio.1002534.s003.docx]

**S2 Table:** **Plasmids.** Unless otherwise referenced, all plasmids were created as part of this study.

| Plasmid | Description | Source |
| --- | --- | --- |
| pAGX2 | P*^ACT1^*-GFP *CEN* *URA3* | [75] |
| pCB591 | GFP-*SEC4 CEN TRP1* |  |
| pCB733 | P*^ACT1^*-GFP-*ABP1* *CEN* *LYS2* |  |
| pCB768 | P*^ACT1^*-GFP-*ABP1* *CEN* *URA3* |  |
| pCB871 | *sec4^Q79L^ HIS3* |  |
| pCB879 | *SLA1*-mRFP:*HIS3-MX6 LEU2 CEN* |  |
| pCB881  pCB901 | *SLA1*-mRFP:*HIS3-MX6 LEU2* 2µ  GFP-*SEC4-Q79L CEN URA3* |  |
| pCB941 | P*^GAL1^*-*SEC4 2*µ *TRP1* |  |
| pCB942 | P*^GAL1^*-*LAS17 2*µ *TRP1* |  |
| pCB954 | pCITE-4a(+) *LAS17-myc* |  |
| pCB964 | GST-*SEC4* |  |
| pCITE-4a(+) | T7-promoter CITE | Novagen, Madison, WI |
| pDD1737 | mRFP:*HIS3-MX6* | D. Drubin, UC, Berkeley |
| pFA6a-GFP-HIS3MX6 | GFP:*HIS3-MX6* | [76] |
| pGEX-4T-1 | GST | GE Healthcare, UK |
| pHVF1-CT | YFP^F1^:*HIS3-MX6* | C. Loewen, UBC |
| pHVF1-NT | *HIS3-MX6*:P*^ACT1^*-YFP^F1^ | C. Loewen, UBC |
| pKT10-GAL-HA | P*^GAL^*-HA 2µ *URA3* | [77] |
| pLC1330 | HA-RFP-*SNC1 CEN URA3* | E. Conibear, UBC |
| pNB810 | *SEC3*-GFP *URA3 CEN* | [78] |
| pPG5-SEC5-3xGFP | *SEC5-*3xGFP *URA3* | [8] |
| pPG5-SEC15-3xGFP | *SEC15-*3xGFP *URA3* | [8] |
| pRC2098 | GFP-*SEC4* *CEN* *URA3* | [13] |
| pRS303 | *HIS3* | [79] |
| pRS426 | 2µ *URA3* | [79] |
| pUVF2-CT | YFP^F2^:*URA3* | C. Loewen, UBC |
| YCplac22 | *CEN TRP1* | [80] |
| YCplac33 | *CEN URA3* | [80] |
| YCplac111 | *CEN LEU2* | [80] |
| YEplac181 | 2µ *LEU2* | [80] |
| YEplac195 | 2µ *URA3* | [80] |
